# Strawberry fields forever: understanding the molecular regulation of strawberry inflorescence architecture

**DOI:** 10.1093/plcell/koag142

**Published:** 2026-05-12

**Authors:** Róisín Fattorini

**Affiliations:** Assistant Features Editor, The Plant Cell, American Society of Plant Biologists, United States; Institute of Molecular Plant Sciences, University of Edinburgh, Edinburgh, United Kingdom

Floral morphology is remarkably diverse and plays a vital role in plant adaptation and reproductive success. Understanding the mechanisms regulating floral development can provide key insight into the evolution of diverse floral forms (Becker et al. 2011). Inflorescences are clusters of flowers arranged on a plant stem, and their morphology is determined, in part, by internode spacing patterns and the arrangement and number of lateral meristems, which form branches and flowers (Prusinkiewicz et al. 2007). Inflorescence architecture, a term encompassing branching patterns and flower number, is an important determinant of agricultural yield. In strawberry (*Fragaria* L.), inflorescence architecture also determines fruit size, with a large primary fruit developing in each inflorescence followed by progressively smaller fruits (Miura et al. 1994). The high variability of strawberry inflorescence architectures and the commercial preference for uniform fruit size mean that unraveling the mechanisms underlying inflorescence development is of both scientific interest and economic value.

In recent work in *The Plant Cell*, Zhenying Zhu and colleagues (Zhu et al. 2026) characterized key genes that regulate the elongation of strawberry inflorescence internodes. An EMS mutant screen produced *basal-branching inflorescence 1* (*bbi1-1* and *bbi1-2*) with perturbed inflorescence morphologies in woodland strawberry (*Fragaria vesca*). Strawberry inflorescence stems include the pedicel that supports each flower and the peduncle that supports the entire inflorescence (Fig. 1a). The *bbi1* mutants had absent or significantly shorter peduncles versus wild-type inflorescences and pedicels that were often longer compared with wild type (Fig. 1b). Two additional allelic mutants, *bbi2-1* and *bbi2-2*, were identified that also exhibited shorter peduncles and longer pedicels than in wild-type plants. Detailed phenotypic analyses of inflorescence development in wild-type, *bbi1*, *bbi2*, and *bbi1/bbi2* plants revealed that wild-type peduncles undergo elongation during early developmental stages, but this does not occur in the mutants. The fully developed peduncles of *bbi* mutants have fewer and shorter cortex cells compared with wild type (Fig. 1c). Interestingly, the converse is true for the pedicels, where *bbi* mutants have a greater number of cortex cells that are longer than wild-type counterparts (Fig. 1d). As such, the inflorescence phenotypes of *bbi* mutants are caused by perturbed cell expansion and cell division in the peduncles and pedicels.

**Figure 1:**
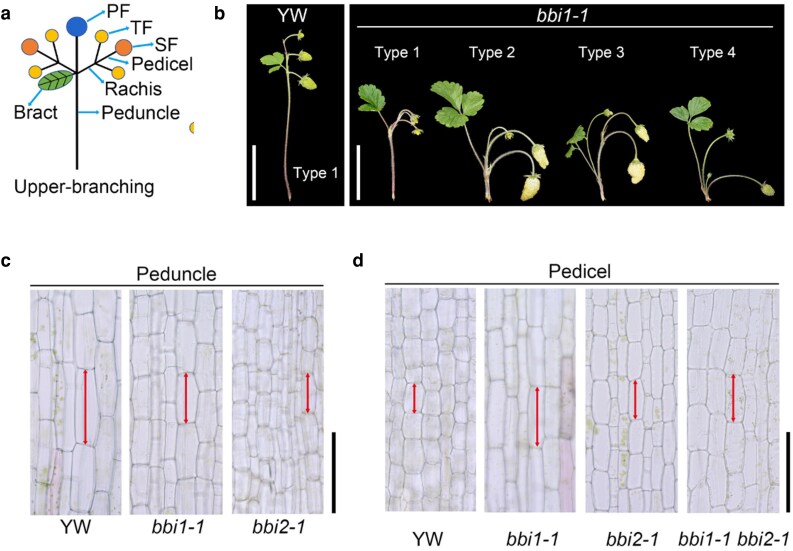
a) Schematic of a woodland strawberry inflorescence with an upper-branching phenotype. b) Inflorescences of wild type (YW) and type 1 to 4 inflorescences of *bbi1-1* plants. Scale bars: 5 cm. c) Longitudinal sections of peduncles from wild type (YW), *bbi1-1*, and *bbi2-1* plants. Scale bar: 100 μm. d) Longitudinal sections of pedicels from wild type (YW), *bbi1-1*, *bbi2-1*, and *bbi1-1 bbi2-1* plants. Scale bar: 100 μm.

Causal mutations were identified for *bbi1* and *bbi2* mutants through a combination of backcrossing experiments and pooled genome resequencing. The causal genes for *bbi1* and *bbi2* encode a G-protein α-subunit (*FveGPA1*) and a brassinosteroid receptor (*FveBRI1*), respectively. The similar mutant phenotypes observed in *bbi2* implied that *bbi1* plants may also have impaired brassinosteroid signaling. This was confirmed through several complementary experiments, including brassinosteroid sensitivity assays, quantification of brassinosteroid metabolites, and analyses of expression levels of genes responsive to brassinosteroid and those involved in its biosynthesis. In addition, plants containing a loss-of-function brassinosteroid-catabolic enzyme (CYP734A129) were crossed with *bbi1-1* mutants. The authors hypothesized that the introduction of the nonfunctional catabolic enzyme should lead to increased brassinosteroid levels and so improve brassinosteroid signal transduction. In these *bbi1-1/cyp734a129* plants, the *bbi1* inflorescence phenotype was indeed largely rescued. As such, *FveGPA1* likely regulates strawberry inflorescence architecture through the brassinosteroid pathway.

In summary, Zhu et al (2026) demonstrate that the brassinosteroid pathway, fine-tuned by heterotrimeric G proteins, plays a pivotal role in regulating inflorescence internode elongation in strawberry. Specifically, FveGPA1 acts through the FveBRI1-mediated pathway to regulate cell division and expansion in inflorescence stems. This regulation occurs in a tissue-specific manner, with elongation suppressed in the pedicels and promoted in the peduncles. Future studies could explore the underlying causes of these tissue-specific patterns and how these genes contribute to the extensive natural variation seen in strawberry inflorescence architecture.

**Recent related articles in *The Plant Cell*:**

• Han et al. (2024) identified an R2R3-MYB transcription factor (FveMYB117a) that represses crown (short branches) outgrowth in strawberry (*Frageria* L.) by inhibiting the accumulation of cytokinin.

• Lu et al. (2024) identified an APETALA2 transcription factor BARE RECEPTACLE required for floral organ initiation in strawberry (*Frageria vesca*). BRE directly binds the GCC-box motif and regulates several auxin pathway genes.

• Li et al. (2025) found that the G-protein Gγ subunit DENSE AND ERECT PANICLE 1 (DEP1) is a positive regulator of brassinosteroid signaling in rice (*Oryza sativa*). They demonstrate how a DEP1-mediated signaling pathway likely activates brassinosteroid responses. This phytohormone is important in regulating plant architecture.

## Data Availability

No new data were generated or analysed in support of this research.
